# Selective expression of neurodegenerative diseases-related mutant p150^Glued^ in midbrain dopaminergic neurons causes progressive degeneration of nigrostriatal pathway

**DOI:** 10.20517/and.2022.07

**Published:** 2022-04-27

**Authors:** Jia Yu, Carmelo Sgobio, Xuan Yang, Yu Peng, Xi Chen, Lixin Sun, Hoon Shim, Huaibin Cai

**Affiliations:** 1 Basic Research Center, Institute for Geriatrics and Rehabilitation, Beijing Geriatric Hospital, Beijing 100095, China.; 2 Transgenic Section, Laboratory of Neurogenetics, National Institute on Aging, National Institutes of Health, Bethesda, MD 20892, USA.; 3 Center for Neuropathology and Prion Research, Ludwig-Maximilians University Munich, Munich 81377, Germany.

**Keywords:** Perry syndrome, motor neuron disease, *DCTN1* gene, dynactin subunit p150^Glued^, missense mutation, nigrostriatal pathway, midbrain dopaminergic neuron, neurodegeneration

## Abstract

**Aim:**

Missense mutations of dynactin subunit p150^Glued^ have been associated with multiple neurodegenerative diseases, including Perry syndrome, characterized by inherited parkinsonism, depression, weight loss, and hypoventilation. The current study investigated how the pathogenic mutant p150^Glued^ affects the integrity and function of the nigrostriatal dopaminergic (DA) pathway *in vivo*.

**Methods:**

Using a tetracycline-controlled transcriptional regulation system, transgenic mouse models were generated with selective overexpression of wild-type, motor neuron disease-related G59S mutant, or Perry syndrome-related G71R mutant human p150^Glued^ in midbrain DA neurons. A series of behavioral, neuropathological, neurochemical, electrochemical, and biochemical studies were performed on the mice to examine and compare the pathogenic impact of the two mutant p150^Glued^ on the survival and function of midbrain DA neurons.

**Results:**

Compared with non-transgenic control mice, transgenic mice overexpressing wild-type human p150^Glued^ showed neither motor phenotypes nor pathological, functional, or biochemical abnormalities of the nigrostriatal DA pathway. Transgenic mice overexpressing G59S mutant p150^Glued^ displayed weight loss, motor deficits, early-onset defects in dopamine transmission, and early-onset loss of DA neurons and axons. Transgenic mice overexpressing G71R p150^Glued^ mutant exhibited hyperactivities, impaired motor coordination, early-onset dysfunction of dopamine uptake, and late-onset loss of DA neurons and axons. In addition, overexpression of either G59S or G71R mutant p150^Glued^ in midbrain DA neurons preferentially downregulated the expression of dopamine transporter at dopaminergic axon terminals. Furthermore, G59S mutant p150^Glued^ rather than G71R mutant p150^Glued^ formed aggregates in midbrain DA neurons *in vivo*, and the aggregates trapped dynein/dynactin, co-localized with lysosomes, and upregulated ubiquitination.

**Conclusion:**

These findings demonstrate that selective expression of either G59S or G71R mutant p150^Glued^ in mouse midbrain DA neurons leads to progressive degeneration of the nigrostriatal DA pathway and indicate that G59S and G71R mutant p150^Glued^ exhibit differential pathogenic impact on the survival and function of midbrain DA neurons *in vivo*.

## INTRODUCTION

Cytoplasmic dynein and its cofactor dynactin mediate microtubule minus-end-directed trafficking, performing critical roles in mitosis, intracellular positioning and movement of organelles, retrograde axonal transport, and synapse formation and stability^[1–3]^. Dynactin is a macromolecular protein complex consisting of more than ten types of subunits, with the largest subunit p150^Glued^ binding directly with dynein and microtubule^[4]^. Missense mutations of *DCTN1*, the gene encoding p150^Glued^, have been linked to different neurodegenerative diseases, in which the G59S missense mutation was linked to autosomal dominant motor neuron disease, and 13 more mutations (F52L, K56R, G67D, K68E, G71A/R/E/V, T72P, Q74P, Y78C/H, and Q93H) were associated with Perry syndrome^[5–8]^. Perry syndrome is an autosomal dominant neurodegenerative disorder characterized by a series of symptoms (parkinsonism, depression, central hypoventilation, and weight loss) and pathology of severe neuronal loss in the *substantia nigra* and *locus coeruleus*^[9,10]^. However, the underlying pathogenic mechanism and potential therapeutic target of neurodegeneration in Perry syndrome remain elusive.

P150^Glued^ contains two microtubule-binding domains (MTBDs) at its N-terminus: the cytoskeleton-associated protein and glycine-rich (CAP-Gly) domain, which interacts with both tubulin and microtubule end binding proteins, and the adjacent serine-rich basic domain, which has an affinity for tubulin^[11,12]^. The MTBDs of p150^Glued^ are required to initiate retrograde transport and maintain microtubule stability in axons^[13–15]^. Deleting the MTBDs in p150^Glued^ upregulates microtubule acetylation, increases the surface level of ionotropic glutamate receptors, disrupts neuromuscular junctions, and leads to selective degeneration of spinal motor neurons^[16]^. Motor neuron disease-related G59S mutation of p150^Glued^ and all the Perry syndrome-related mutations are point mutations clustered within or close to the CAP-Gly domain, resulting in loss-of-function of the MTBD^[6–8]^. Furthermore, G59S mutant p150^Glued^, as well as G71A, G71E, G71R, T72P, Q74P, and F52L mutant p150^Glued^, form cytoplasmic aggregates *in vitro* in a time- and concentration-dependent manner^[17–19]^, suggesting both loss-of-function and toxic-gain-of-function mechanisms might be involved in *DCTN1* mutation-induced neurodegeneration. Additionally, G59S mutant p150^Glued^ is less stable^[20]^, and both G59S and Perry syndrome-related mutant p150^Glued^ compromise normal functions of dynactin p150^Glued^ in a dominant-negative fashion when overexpressed^[13,14,21]^, suggesting a dominant-negative mechanism for *DCTN1* mutation-induced neurodegeneration. Movement disorder and the underlying neuropathology, including loss of spinal motor neurons and degeneration of axon and neuromuscular junctions, have been reported in G59S mutant p150^Glued^ knock-in and transgenic mice^[20–22]^. Behavioral defects have also been found in G71A mutant p150^Glued^ knock-in and transgenic mice^[23,24]^. However, how the pathogenic mutant p150^Glued^ affects the integrity and function of the nigrostriatal DA pathway has not been critically examined in animal models.

Here, we investigated and compared the pathogenic impact of two different neurodegenerative diseases-related mutant p150^Glued^ in the survival and function of midbrain DA neurons *in vivo*. Transgenic mouse models with selective overexpression of wild-type, G59S mutant, or G71R mutant human p150^Glued^ in midbrain DA neurons were generated using a tetracycline-controlled transcriptional regulation system. A series of experiments were performed to identify any behavioral, neuropathological, neurochemical, electrochemical, and biochemical abnormalities in the transgenic mice. Our findings highlight the importance of p150^Glued^ and its MTBDs in maintaining the survival and function of midbrain DA neurons and reveal the pathogenic similarity and difference between G59S and G71R mutant 150^Glued^.

## METHODS

### Animals

The *tetO*-human *DCTN1* WT transgenic mice (for simplicity, referred to as tetO-WT) carrying the wild-type (WT) human p150^Glued^ gene (*DCTN1*) under the control of tetracycline operator (*tetO*) were generated as described previously^[25,26]^. The tetO-WT mice were mated with *Pitx3-IRE2-tTA* mice (referred to as tTA, a “Tet-Off” tool, JAX stock #021962), which predominantly express tetracycline-controlled transactivator protein (tTA) in midbrain DA neurons^[27]^. This breeding strategy produced *Pitx3-IRE2-tTA/tetO*-human *DCTN1* WT transgenic mice (referred to as WT Tg), which had the selective expression of wild-type human p150^Glued^ in midbrain DA neurons, and littermate controls [non-transgenic (nTg), tetO-WT, and *tTA* mice].

Following the same protocol, *Pitx3-IRE2-tTA/tetO*-human *DCTN1* G59S transgenic mice (referred to as G59S Tg), which had the selective expression of G59S mutant human p150^Glued^ in midbrain DA neurons, and littermate controls (nTg, tetO-G59S, and *tTA* mice) were obtained.

Following the same protocol, *Pitx3-IRE2-tTA/tetO*-human *DCTN1* G71R transgenic mice (referred to as G71R Tg), which had the selective expression of G71R mutant human p150^Glued^ in midbrain DA neurons, and littermate controls (nTg, tetO-G71R, and tTA mice) were obtained.

The mice were housed in a 12 h light/dark cycle and fed a regular diet *ad libitum*. All mouse work followed the guidelines approved by the Institutional Animal Care and Use Committees of National Institute on Aging, NIH.

### Genotyping

Genomic DNA was extracted from tail biopsy using DirectPCR Lysis Reagent (Viagen Biotech) and used for PCR amplification. Genotyping was performed with specific sets of PCR primers for *Pitx3-IRES2-tTA* transgene (GACTGGCTTGCCCTCGTCCCA and GTGCACCGAGGCCCCAGATCA)^[27]^ and *tetO-*human *DCTN1* transgene (AGGAGATGGCTGATACTGCTGATG and TTCATCGCTTCCAAGTCTCCCACA).

### Behavior tests

Cohorts of *WT Tg* mice and littermate controls [nTg (*n* = 10), tTA (*n* = 10), tetO-WT (*n* = 10), and WT Tg (*n* = 10)] were repeatedly weighed and assessed for motor function at 1, 6, 12, and 18 months of age.

Cohorts of *G59S Tg* mice and littermate controls [nTg (*n* = 10), tTA (*n* = 11), tetO-G59S (*n* = 10), and G59S Tg (*n* = 10)] were repeatedly weighed and assessed for motor function at 1, 6, 12, and 18 months of age.

Cohorts of *G71R Tg* mice and littermate controls [nTg (*n* = 11), tTA (*n* = 10), tetO-G71R (*n* = 10), and G71R Tg (*n* = 10)] were repeatedly weighed and assessed for motor function at 1, 6, 12, and 18 months of age.

#### Open-field test.

Mice were placed in the open-field apparatus of the Flex-Field Activity System (San Diego Instruments). Ambulatory, rearing, and fine movements of mice were traced and quantified with infrared photobeams sensors and Flex-Field software in the unit as the number of beam breaks per 30 min.

#### Rotarod test.

Mice were placed onto a rotating rod with auto-acceleration from 0 to 40 rpm within 1 min (San Diego Instruments). The time length that each mouse stayed on the rotating rod was measured across three trials.

### Histology, immunostaining, and light microscopy

Mice were sacrificed and perfused with 4% paraformaldehyde (PFA) in phosphate-buffered saline (PBS). Mouse brains were dissected, post-fixed in 4% PFA/PBS solution overnight, and submerged in 30% sucrose in PBS for at least 72 h. The 40 μm-thick brain sections were prepared using CM1950 cryostat (Leica) and stained with antibodies specific to human and mouse p150^Glued^ (amino acid 3–202 at the N-terminus of p150^Glued^, 1:200, BD Biosciences, Catalog # 610474, recognizing wild-type p150^Glued^ but not G59S mutant p150^Glued^, G71R mutant p150^Glued^, or p135+), human and mouse p150^Glued^ and p135+ (amino acid 1266–1278 at the C-terminus of p150^Glued^ and p135+, 1:500, Abcam, Catalog # ab11806, recognizing wild-type, G59S mutant and G71R mutant p150^Glued^, and p135+), tyrosine hydroxylase (TH, 1:2500, Pel-freez, Catalog # P40101–150; 1:500, ImmunoStar, Catalog # 22941), dynactin subunit p50 (1:1000, Millipore, Catalog # AB5869P), dynactin subunit actin-related protein 1 (ARP1, 1:1000, Millipore, Catalog # AB6058), cytoplasmic dynein light intermediate chain (DLIC, 1:500, Abcam, Catalog # ab123901), lysosomal associated membrane protein 2 (LAMP2, 1:500, Abcam, Catalog # ab13524), and ubiquitin (1:500, Enzo Life Sciences, Catalog # BML-PW0930), as suggested by manufacturers. Fluorescent signals were visualized with Alex Fluor-conjugated secondary antibody (1:500, Invitrogen, Catalog # A-11055, A-21202, A-21206, A-21208, A-21447, A10036, and A10040) and examined using LSM 880 laser-scanning confocal microscope (Zeiss). The paired images in all figures were collected using identical gain and offset settings, processed uniformly, and displayed as either a single optic layer or the maximum-intensity projections of the z-series confocal stack at 1.0 μm intervals.

### Stereology

The number of midbrain dopaminergic neurons was estimated by unbiased stereology, as described previously^[27,28]^. According to Paxinos and Franklin’s The Mouse Brain in Stereotaxic Coordinates (4th edition), a series of coronal sections (40 μm thick) across the midbrain (every fourth section from Bregma −2.54 to −4.24 mm, 10 sections per animal) were stained with an antibody specific to TH (1:2500, Pel-Freez, Catalog # P40101–150). After subsequent staining with Vectastain Elite ABC Kit, sections were visualized with DAB Kit (Vector Laboratories). Bright-field images were captured, and the number of TH-positive neurons was assessed using the optical fractionator function of Stereo Investigator 10 (MicroBrightField) under the Axio microscope Imager A1 (Zeiss). Counters were blinded to the genotypes of mice, and the sampling scheme was designed to have a coefficient of error less than 10% for the reliability of results.

### Image analysis

Quantitative analysis of images was performed with ImageJ (NIH). After converting image type to 8-bit, areas of interest were selected with Freehand tools, and then mean optical intensities or area fractions were measured. The mean intensity for the background area was subtracted from the selected area to determine the net mean intensity. For quantitative analysis of DA neurite dystrophy, 5 tile images per animal (5 sections per animal and 1 tile image per section) from the *substantia nigra pars reticulata* (SNr) were taken with a 20× lens. Dystrophic DA neurites were defined as neuritic varicosity ≥ 25 μm^2^. The area of dystrophic TH-positive DA neurites and the area of the SNr were quantified with ImageJ (NIH). For quantitative analysis of DA axon degeneration, 50 images per animal (10 sections per animal and 5 images per section) from the dorsal striatum were taken with a 63× lens. The area fraction of TH-positive DA axon terminals in each image was quantified with ImageJ (NIH). For quantitative analysis of G59S mutation-induced p150^Glued^ aggregates, aggregates were defined as puncta ≥ 1 μm^2^.

### High-performance liquid chromatography (HPLC)

The dorsal striatum of mice was dissected, weighed, and homogenized by sonication in 0.1 N perchloric acid containing 100 μM EDTA (500 μl buffer per 100 mg tissue). After centrifugation, the supernatant was collected and assayed for dopamine and DOPAC content by HPLC with electrochemical detection, as described previously^[27,28]^. Briefly, the mobile-phase solution containing octanesulfonic acid was used as an ion-pairing agent and pumped isocratically through a reversed-phase liquid chromatographic column. DA and DOPAC were measured by the current produced after exposure of the eluate to a flow-through electrode set to oxidizing and then reducing potentials in series, with recordings from the last electrode reflecting reversibly oxidized species.

### Fast-scan cyclic voltammetry (FSCV)

The kinetics of striatal dopamine release evoked by electrical stimulation was measured using FSCV, as described previously^[27,28]^. The 400 μm-thick slices of dorsal striatum were prepared and transferred into 32 °C oxygenated artificial cerebrospinal fluid (aCSF: 126 mM NaCl, 2.5 mM KCl, 1.2 mM NaH_2_PO_4_, 2.4 mM CaCl_2_, 1.2 mM MgCl_2_, 25 mM NaHCO_3_, 11 mM glucose, 20 mM 4-(2-hydroxyethyl)-1-piperazineethanesulfonic acid, and 0.4 mM L-ascorbic acid). Cylindrical carbon-fiber microelectrode prepared with T650 fibers (Goodfellow) was held at −0.4 V, and the potential was increased to 1.2 V and back at 400 V/s every 100 ms using a triangle waveform. Dopamine release in striatal slices was evoked by rectangular, electrical pulse stimulation (100–400 μA, 0.6 ms per phase, biphasic) applied every 5 min. Data were collected and analyzed with the Demon Voltammetry and Analysis software suite^[29]^. Ten cyclic voltammograms of charging currents were recorded as background before stimulation, and the average of these responses was subtracted from data obtained during and after stimulation. Maximum amplitudes of extracellular dopamine transients were calculated from input/output function (I*/*O) curves. I*/*O curves were plotted with stimulus current against the concentration of dopamine response amplitude over a range of stimulus intensities. The time constant of the slope decay (τ) was used as an uptake kinetic indicator of evoked dopamine release. Following experiments, electrodes were calibrated using 1 and 10 μM dopamine solutions in aCSF.

### Western blot analysis

Mouse brain tissues were homogenized and sonicated in 1% SDS lysis buffer (50 mM Tris-HCl, 150 mM NaCl, 2 mM EDTA, pH 7.5, and 1% SDS) supplemented with Protease/Phosphatase Inhibitor Cocktails (ThermoFisher Scientific). Lysates were centrifuged at 15,000 *g* for 15 min at 4 °C. Supernatants were collected, and protein content was quantified with the bicinchoninic acid (BCA) assay kit (ThermoFisher Scientific). Equal amounts of total proteins were size-fractioned with NuPage 4%−12% Bis-Tris gel in MES or MOPS running buffer (ThermoFisher Scientific). After transferring to nitrocellulose membranes with the Trans-Blot Turbo Transfer System (Bio-Rad), proteins were immunoblotted with specific primary antibodies to human p150^Glued^ (1:1000, Covance, custom-made), human and mouse p150^Glued^ and p135+ (1:1000, Abcam, Catalog # ab11806), dynactin subunit DCTN4 (1:1000, Abcam, Catalog # ab170107), dynactin subunit p50 (1:1000, BD Biosciences, Catalog # 611002), dynactin subunit actin-related protein 1 (ARP1, 1:1000, Sigma-Aldrich, Catalog # A5601), tyrosine hydroxylase (TH, 1:1000, Sigma-Aldrich, Catalog # T1299), dopamine transporter (DAT, 1:1000, Millipore, Catalog # MAB369), and β-actin (1:5000, Sigma-Aldrich, Catalog # A1978). Protein signals were visualized using the Odyssey System with IRDye secondary antibodies (LI-COR Biosciences, Catalog # 926–32212, 926–32219, 926–3221, 926–32411, and 926–68023) and quantified with NIH ImageJ software.

### Statistical analysis

Statistical analysis was performed using GraphPad Prism 9 (GraphPad Software). Data are presented as mean ± SEM. Statistical significance was determined by comparing means of different groups using unpaired t-test, one-way, and two-way ANOVA with post hoc analysis. *P < 0.05, **P < 0.01, ***P < 0.001, ****P < 0.0001.

## RESULTS

### Generation of mouse models with selective expression of wild-type or mutant human p150^Glued^ in midbrain DA neurons

**Figure 1.**
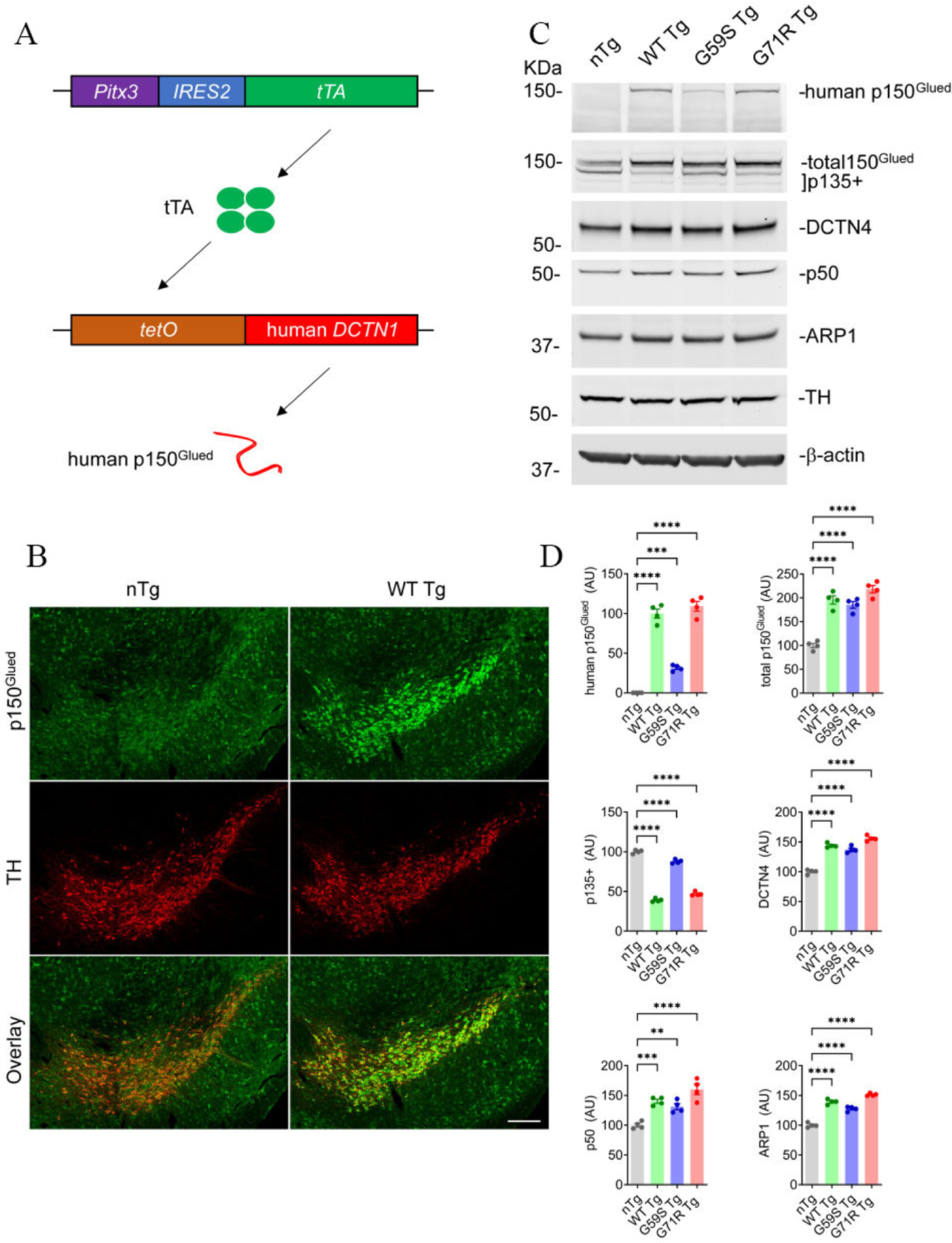
Selective expression of wild-type or mutant human p150^Glued^ in mouse midbrain DA neurons. (A) The schematic diagram depicts the generation of transgenic mice with target expression of human p150^Glued^ in midbrain DA neurons by crossing *Pitx3-IRE2-tTA* and *tetO*-human *DCTN1* mice. (B) Immunofluorescent images show the staining of p150^Glued^ (green) and TH (red) in the coronal midbrain sections of one-month-old *nTg* and *WT Tg* mice. DA neurons were visualized by TH staining. Scale bar: 200 μm. (C, D) Western blots show the expression of human p150^Glued^, total (human and mouse) p150^Glued^, p135+, DCTN4, p50, and ARP1 in the midbrain homogenates of one-month-old *nTg*, *WT Tg*, *G59S Tg*, and *G71R Tg* mice. β-actin and TH were used as the loading control. The bar graph estimates the protein level of dynactin subunits normalized with β-actin (*n* = 4 per genotype). Data are presented as mean ± SEM. One-way ANOVA with Dunnett’s multiple comparisons test was used for statistical analysis (the mean of each genotype was compared with the mean of nTg). **P < 0.01, ***P < 0.001, ****P < 0.0001.

To investigate the involvement and mechanism of p150^Glued^ in neurodegeneration *in vivo*, three lines of *tetO-*human *DCTN1* mice (tetO-WT, tetO-G59S, or tetO-G71R) with inducible expression of wild-type or mutant human p150^Glued^ under the transcriptional control of tetracycline operator (*tetO*) were generated. The *tetO*-human *DCTN1* mice were then crossed with *Pitx3-IRE2-tTA* knock-in mice to obtain *Pitx3-IRE2-tTA/tetO*-human *DCTN1* WT (WT Tg), *Pitx3-IRE2-tTA/tetO*-human *DCTN1* G59S (G59S Tg), or *Pitx3-IRE2-tTA/tetO*-human *DCTN1* G71R (G71R Tg) bigenic mice [Figure 1A]. Since the tetracycline transactivator (tTA) is specifically expressed in midbrain DA neurons under the endogenous *Pitx3* promoter^[27]^, it turned on the expression of human wild-type or mutant p150^Glued^ selectively in midbrain DA neurons [Figure 1A]. The target overexpression of human p150^Glued^ in midbrain DA neurons of one-month-old WT mice was visualized by immunostaining [Figure 1B]. Using a human p150^Glued^-specific antibody, Western blot confirmed the expression of human p150^Glued^ in the midbrain homogenates of one-month-old *WT Tg*, *G59S Tg*, and *G71R Tg* mice but not *nTg* mice [Figure 1C and D]. The steady level of G59S human p150^Glued^ was significantly less (approximately 30%) compared with WT or G71R human p150^Glued^. Western blot further revealed substantially increased levels of total (human and mouse) p150^Glued^ (approximately two-fold) and other dynactin subunits, including DCTN4, p50, and ARP1, but markedly reduced level of p135+ (alternative splicing isoforms of *Dctn1* lacking the N-terminus) in the midbrain homogenates of *WT Tg*, *G59S Tg*, and *G71R Tg* mice compared to the *nTg* mice [Figure 1C and D].

### *G59S Tg* and *G71R Tg* mice exhibit profound motor abnormalities.

**Figure 2.**
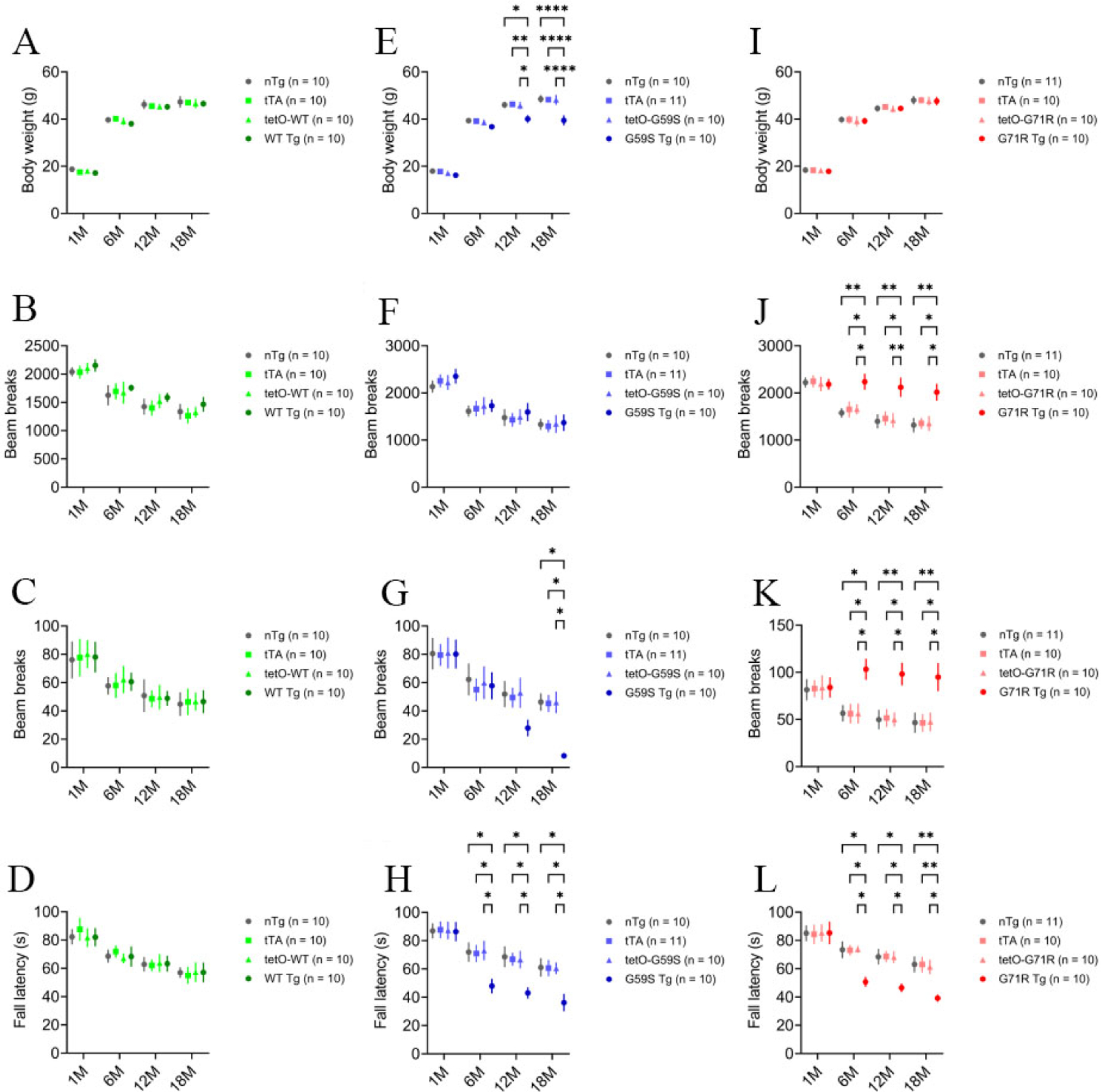
Profound motor abnormalities of *G59S Tg* and *G71R Tg* mice. (A-D) At 1, 6, 12, and 18 months of age, cohorts of male *nTg*, *tTA*, *tetO-WT*, and *WT Tg* mice (*n* = 10 per genotype) were repeatedly weighed (A); tested for ambulatory movement (B) and rearing (C) in the open-field test; and examined for the latency to fall in the rotarod test (D). (E-H) At 1, 6, 12, and 18 months of age, cohorts of male *nTg* (*n* = 10), *tTA* (*n* = 11), *tetO-G59S* (*n* = 10), and *G59S Tg* (*n* = 10) mice were repeatedly weighed (E); tested for ambulatory movement (F) and rearing (G) in the open-field test; and examined for the latency to fall in the rotarod test (H). (I-L) At 1, 6, 12, and 18 months of age, cohorts of male *nTg* (*n* = 11), *tTA* (*n* = 10), *tetO-G71R* (*n* = 10), and *G71R Tg* (*n* = 10) mice were repeatedly weighed (I); tested for ambulatory movement (J) and rearing (K) in the open-field test; and examined for the latency to fall in the rotarod test (L). Data are presented as mean ± SEM. Two-way ANOVA with Tukey’s multiple comparisons test was used for statistical analysis (at each time point, the mean of each genotype was compared with the mean of every other genotype). *P < 0.05, **P < 0.01, ****P < 0.0001.

As motor symptoms and weight loss are the main clinical manifestations in familial motor neuron disease and Perry syndrome cases with *DCTN1* mutation^[5]^, *WT Tg*, *G59S Tg*, *G71R Tg* mice, and their littermate controls were weighed, tested for locomotion activity in the open-field test, and examined for motor coordination in the rotarod test at 1, 6, 12, and 18 months of age. *WT Tg* mice developed normally and exhibited comparable body weight and motor function with littermate single- or non-transgenic mice in the open field test and the rotarod test [Figure 2A–D]. *G59S Tg* mice displayed substantial weight loss starting at 12 months of age, normal ambulatory movement, dramatic reduction of rearing movement at 18 months of age, and significant impairment in rotarod test starting at six months of age [Figure 2E–H]. *G71R Tg* mice showed normal body weight, significant hyperactivities of ambulatory movement and rearing, and substantially impaired rotarod performance starting at six months of age [Figure 2I–L]. Together, these findings demonstrate that selective expression of either G59S or G71R mutant p150^Glued^ in midbrain DA neurons causes profound motor abnormalities in mice and suggest that both G59S and G71R mutant p150^Glued^ may impair the function and survival of midbrain DA neurons.

### *G59S Tg* and *G71R Tg* mice develop progressive degeneration of midbrain DA neurons

**Figure 3.**
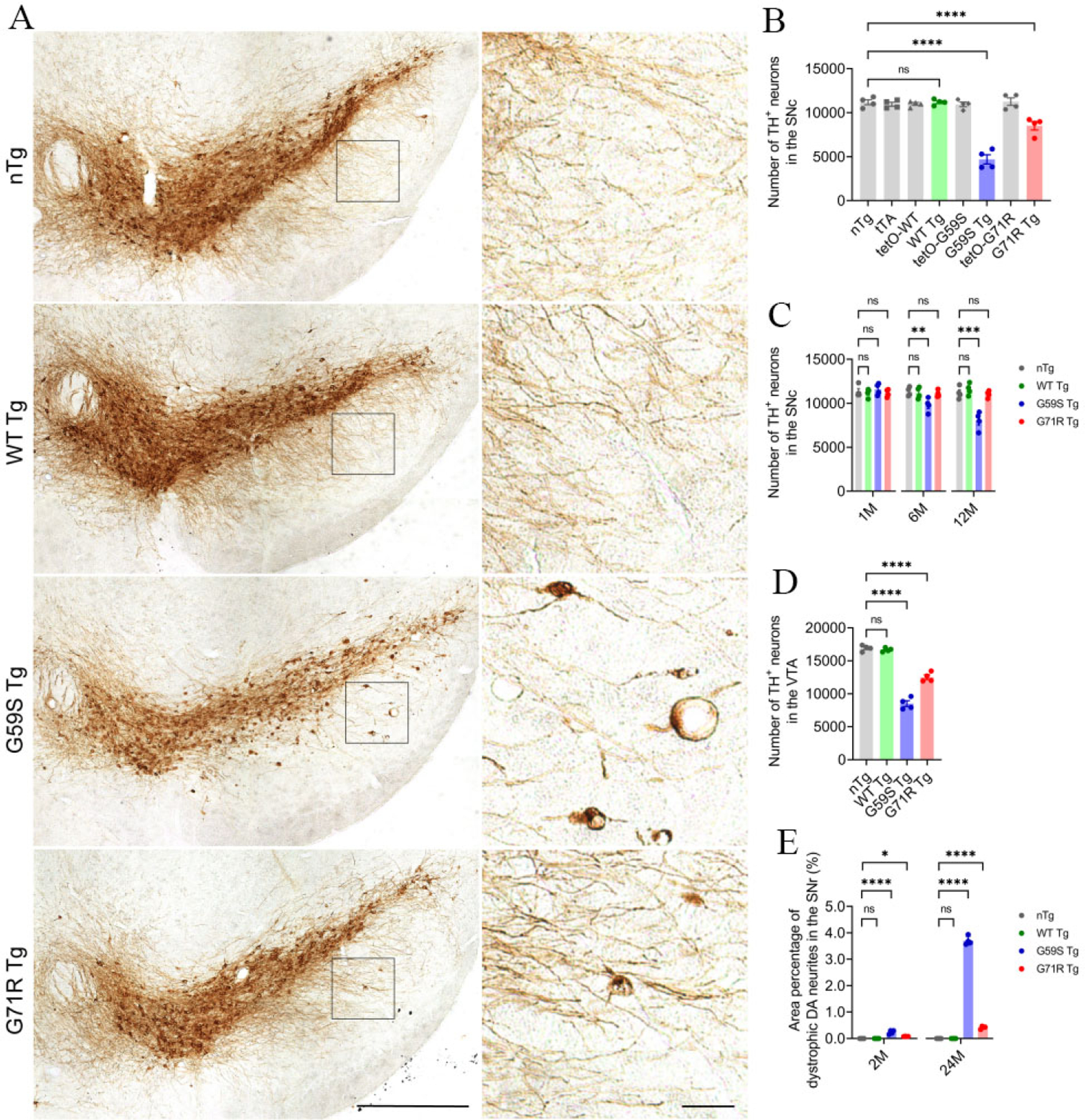
Progressive degeneration of DA neurons in the midbrain of *G59S Tg* and *G71R Tg* mice. (A) Immunohistochemical images show TH staining in the coronal midbrain sections of 24-month-old *nTg*, *WT Tg*, *G59S Tg*, and G71R Tg mice. Scale bar: (left) 500 μm (low-magnification images) and (right) 50 μm (high-magnification images). Note the dystrophic TH-positive DA neurites in the SNr of *G59S Tg* and *G71R Tg* mice. (B) Bar graphs depict the unbiased stereological estimation of the number of TH-positive DA neurons in the SNc of 24-month-old *nTg*, *tTA, tetO-WT*, *WT Tg*, *tetO-G59S*, *G59S Tg*, *tetO-G71R*, *and G71R Tg* mice (*n* = 4 per genotype). Data are presented as mean ± SEM. One-way ANOVA with Dunnett’s multiple comparisons test was used for statistical analysis (the mean of each genotype was compared with the mean of nTg). ****P < 0.0001. ns: Not significant. (C) Bar graphs depict the unbiased stereological estimation of the number of TH-positive DA neurons in the SNc of 1-, 6-, and 12-month-old *nTg*, *WT Tg*, *G59S Tg*, and *G71R Tg* mice (*n* = 4 per genotype per time point). Data are presented as mean ± SEM. At each time point, one-way ANOVA with Dunnett’s multiple comparisons test was used for statistical analysis (the mean of each genotype was compared with the mean of nTg). **P = 0.0099, ***P = 0.0001. (D) Bar graphs depict the unbiased stereological estimation of the number of TH-positive DA neurons in the VTA of 24-month-old *nTg*, *WT Tg*, *G59S Tg*, *and G71R Tg* mice (*n* = 4 per genotype). Data are presented as mean ± SEM. One-way ANOVA with Dunnett’s multiple comparisons test was used for statistical analysis (the mean of each genotype was compared with the mean of nTg). ****P < 0.0001. (E) Bar graphs quantify the area percentage of dystrophic TH-positive DA neurites in the SNr of 2- and 24-month-old *nTg*, *WT Tg*, *G59S Tg*, *and G71R Tg* mice (at each time point, *n* = 4 animals per genotype and 5 sections per animal). Dystrophic DA neurites were defined as neuritic varicosity ≥ 25 μm^2^. Data are presented as mean ± SEM. At each time point, one-way ANOVA with Dunnett’s multiple comparisons test was used for statistical analysis (the mean of each genotype was compared with the mean of nTg). *P = 0.0259, ****P < 0.0001. TH: Tyrosine hydroxylase.

Since neuronal loss in *substantia nigra* is a pathological hallmark of Perry syndrome^[9,10]^, the numbers of TH-positive midbrain DA neurons in the *substantia nigra pars compacta* (SNc) and ventral tegmental area (VTA) of *WT Tg*, *G59S Tg*, *G71R Tg* mice, and their control mice were counted using an unbiased stereological approach. *WT Tg* mice presented with no apparent loss of DA neurons in either the SNc or VTA at 1, 6, 12, and 24 months of age compared with control mice [Figure 3A–D]. *G59S Tg* mice displayed severe and early-onset degeneration of DA neurons in the midbrain, with approximately 13%, 28%, and 57% neuronal loss in the SNc at 6, 12, and 24 months of age, respectively, and 50% of neuronal loss in the VTA at 24 months of age [Figure 3A–D]. *G71R Tg* mice exhibited mild and late-onset degeneration of DA neurons in the midbrain, with approximately 25% neuronal loss in the SNc as well as the VTA at 24 months of age [Figure 3A–D]. Interestingly, besides neuronal loss, *G59S Tg* and *G71R Tg* mice showed remarked and progressive dystrophy of DA neurites in the *substantia nigra pars reticulata* (SNr) starting at two months of age [Figure 3A and E]. Therefore, expression of either G59S or G71R mutant p150^Glued^ in midbrain DA neurons leads to substantial and progressive neurodegeneration.

### *G59S Tg* and *G71R Tg* mice display robust degeneration of DA axon terminals

**Figure 4.**
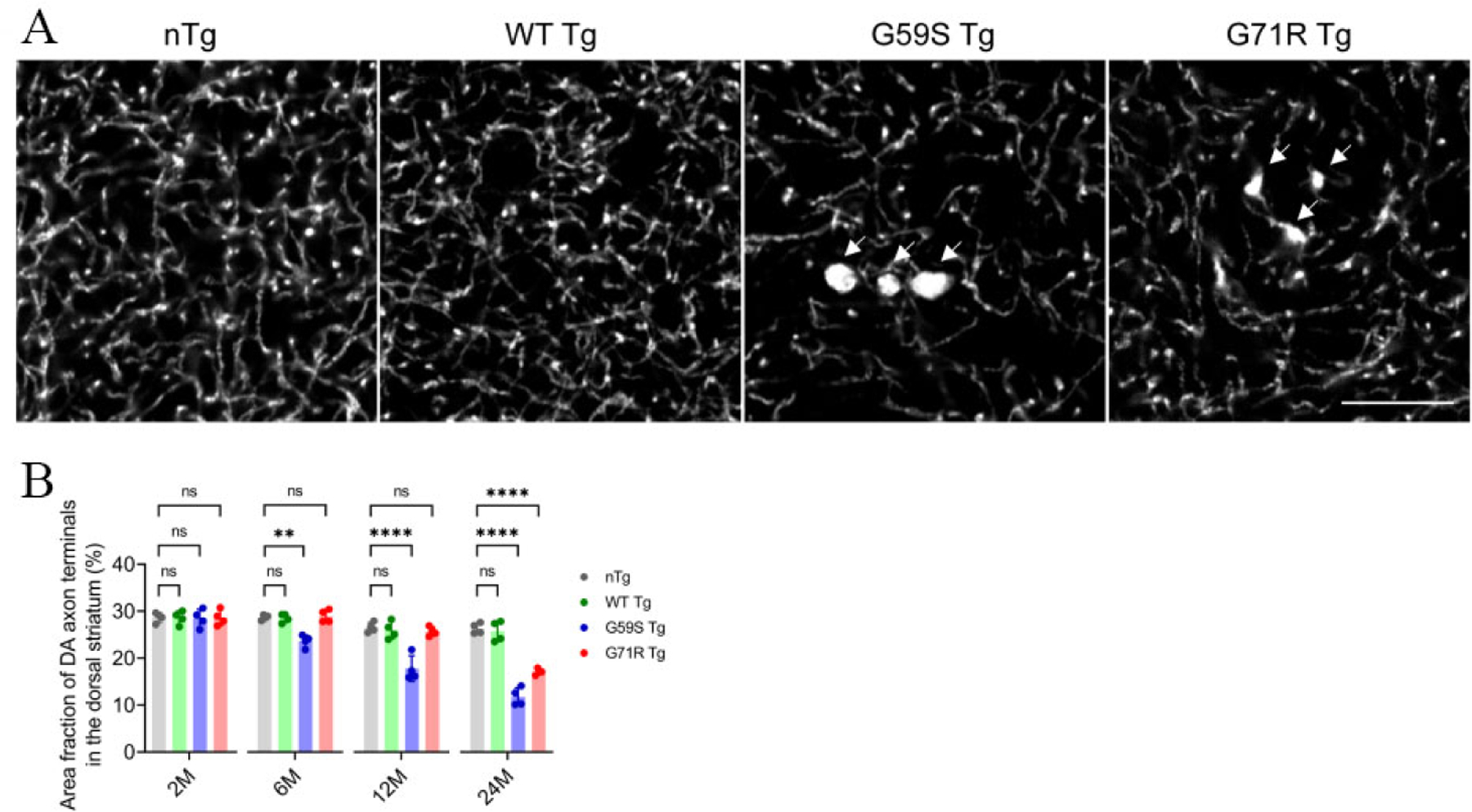
Robust degeneration of DA axon terminals in the striatum of *G59S Tg* and *G71R Tg* mice. (A) Representative images show TH staining in the dorsal striatum coronal sections of 24-month-old *nTg*, *WT Tg*, *G59S Tg*, and *G71R Tg* mice. Scale bar: 10 μm. Arrows point to swellings of TH-positive DA axon terminals in the striatum of *G59S Tg* and *G71R Tg* mice. (B) Bar graph estimates the area fraction of TH-positive DA axon terminals in the dorsal striatum of 2-, 6-, 12-, and 24-month-old *nTg*, *WT Tg*, *G59S Tg*, and *G71R Tg* mice (at each time point, *n* = 4 animals per genotype and 10 sections per animal). Data are presented as mean ± SEM. At each time point, one-way ANOVA with Dunnett’s multiple comparisons test was used for statistical analysis (the mean of each genotype was compared with the mean of nTg). **P = 0.0018, ****P < 0.0001. ns: Not significant. TH: Tyrosine hydroxylase.

As axon dystrophy was reported in the striatum of patients with Perry syndrome^[30,31]^, the density and morphology of DA axon terminals in the dorsal striatum of *nTg*, *WT Tg*, *G59S Tg*, and *G71R Tg* mice were examined. Compared with age-matched nTg controls, 2-, 12-, and 24-months old *WT Tg* mice had no apparent alteration of TH-positive DA axon terminals [Figure 4A and B]. However, *G59S Tg* and *G71R Tg* mice displayed substantial loss of DA axon terminals, with approximately 15%, 33%, and 55% of axonal loss in 6-, 12- and 24-month-old *G59S Tg* mice, respectively, and 35% of axonal loss in 24-month-old *G71R Tg* mice [Figure 4A and B]. Furthermore, TH staining also revealed abnormally enlarged DA axon terminals in the striatum of *G59S Tg* and *G71R Tg* mice [Figure 4A, arrow]. These data demonstrate that expression of either G59S or G71R mutant p150^Glued^ in midbrain DA neurons causes robust degeneration of DA axon terminals and suggest dysfunctional DA transmission in *G59S Tg* and *G71R Tg* mice.

### G59S Tg and G71R Tg mice show alteration of dopamine transmission

**Figure 5.**
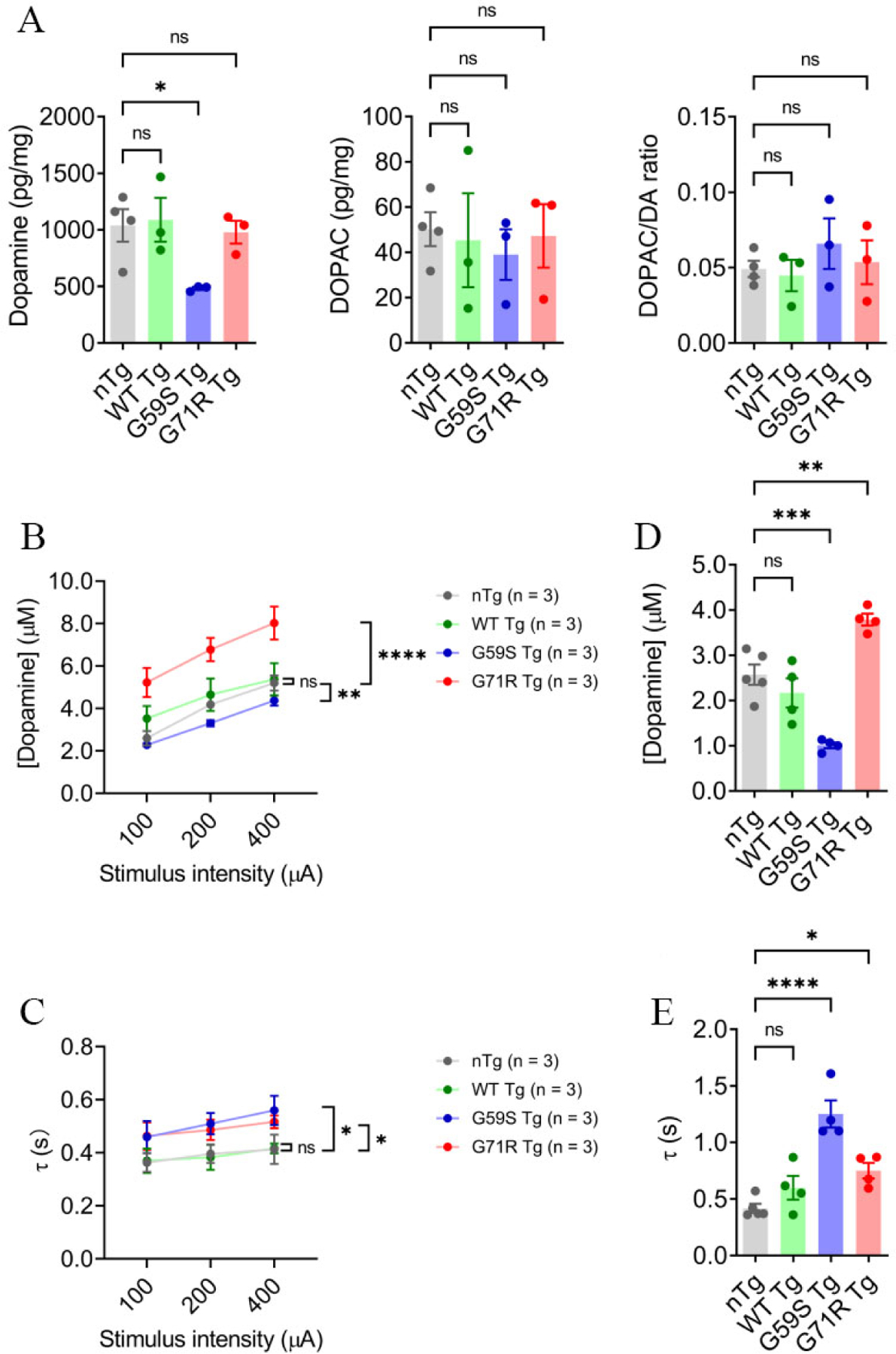
Alteration of dopamine content and release in the striatum of *G59S Tg* and *G71R Tg* mice. (A) HPLC measures the content of dopamine and DOPAC in the dorsal striatum of 18-month-old *nTg*, *WT Tg*, *G59S Tg*, and *G71R Tg* mice (*n* = 3 or 4 per genotype). Data are presented as mean ± SEM. One-way ANOVA with Dunnett’s multiple comparisons test was used for statistical analysis (the mean of each genotype was compared with the mean of nTg). *P = 0.0397. ns: Not significant. (B, C) FSCV quantifies the peak evoked dopamine release (B) and time constant of slope decay (C) following single-pulse electrical stimulation of different stimulus intensity in the dorsal striatum of six-month-old *nTg*, *WT Tg*, *G59S Tg*, *and G71R Tg* mice (*n* = 3 animals per genotype and 3 sections per animal). Data are presented as mean ± SEM. Two-way ANOVA genotype factor: nTg *vs*. G59S Tg, *F*_(1,12)_ = 25.11, **P = 0.0044; nTg *vs*. G71R Tg, *F*_(1,12)_ = 40.19, ****P < 0.0001 (B). Two-way ANOVA genotype factor: nTg *vs*. G59S Tg, *F*_(1,12)_ = 9.285, *P = 0.0101; nTg *vs*. G71R Tg, *F*_(1,12)_ = 8.491, *P = 0.0130 (C). (D,E) FSCV quantifies the peak evoked dopamine release (D) and time constant of slope decay (E) in response to burst electrical stimulation (200 μA, 50 Hz, 5 pulses) in the dorsal striatum of 18-month-old *nTg*, *WT Tg*, *G59S Tg*, and *G71R Tg* mice (n = 4 or 5 animals per genotype and 3 sections per animal). Data are presented as mean ± SEM. One-way ANOVA with Dunnett’s multiple comparisons test was used for statistical analysis (the mean of each genotype was compared with the mean of nTg). nTg *vs*. G59S Tg, ***P = 0.0004; nTg *vs*. G71R Tg, **P = 0.0034 (D). nTg *vs*. G59S Tg, ****P < 0.0001; nTg *vs*. G71R Tg, *P = 0.0357 (E).

Considering the pathological findings, the steady-state level of dopamine in the dorsal striatum of *nTg*, *WT Tg*, *G59S Tg*, and *G71R Tg* mice was assessed with HPLC. At 18 months of age, *WT Tg* and *G71R Tg* mice showed no apparent change in dopamine level compared with nTg controls, while *G59S Tg* mice displayed a significant reduction of dopamine content, with an approximately 53% of decrease [Figure 5A]. Regarding the primary dopamine metabolite 3,4-dihydroxyphenylacetic acid (DOPAC), neither its content nor the DOPAC/dopamine ratio in 18-month-old *WT Tg*, *G59S Tg*, and *G71R Tg* mice was significantly different with age-matched nTg controls [Figure 5A]. These data reveal dopamine deficiency in the striatum of *G59S Tg* mice, which might be the functional consequence of robust degeneration of midbrain DA neurons and the pathophysiological basis for the motor deficits observed in *G59S Tg* mice. By contrast, at 18 months of age, *G71R Tg* mice developed neither loss of midbrain DA neurons nor reduced dopamine content, further supporting the late-onset DA neuronal loss in *G71R Tg* mice.

Next, dopamine release kinetics in the dorsal striatum of *nTg*, *WT Tg*, *G59S Tg*, and *G71R Tg* mice was evaluated using fast-scan cyclic voltammetry (FSCV). At six months of age, *WT Tg* mice showed comparable evoked dopamine release and dopamine uptake in response to single-pulse stimulation compared with nTg controls [Figure 5B and C]. However, *G59S Tg* mice displayed significantly decreased dopamine release and slower dopamine uptake, while *G71R Tg* mice exhibited substantially increased dopamine release and slower dopamine uptake [Figure 5B and C]. Similarly, 18-month-old *WT Tg* mice showed comparable evoked dopamine release and dopamine uptake in response to burst-pulse stimulation compared with age-matched nTg controls [Figure 5D and E]. However, *G59S Tg* mice displayed significantly decreased dopamine release and slower dopamine uptake, while *G71R Tg* mice exhibited substantially increased dopamine release and slower dopamine uptake [Figure 5D and E]. Taken together, *G59S Tg* mice showed early-onset defects in striatal dopamine transmission, consistent with the early-onset loss of DA neurons and axons. In contrast, *G71R Tg* mice showed early-onset dysfunction of dopamine release and uptake long before the substantial loss of DA neurons and axons occurred. Thus, these data indicate that expression of either G59S or G71R mutant p150^Glued^ in midbrain DA neurons profoundly compromises the dopamine transmission of the nigrostriatal DA pathway, contributing to the motor phenotypes developed in these transgenic mice.

### G59S Tg and G71R Tg mice exhibit an age-dependent reduction of DA proteins

**Figure 6.**
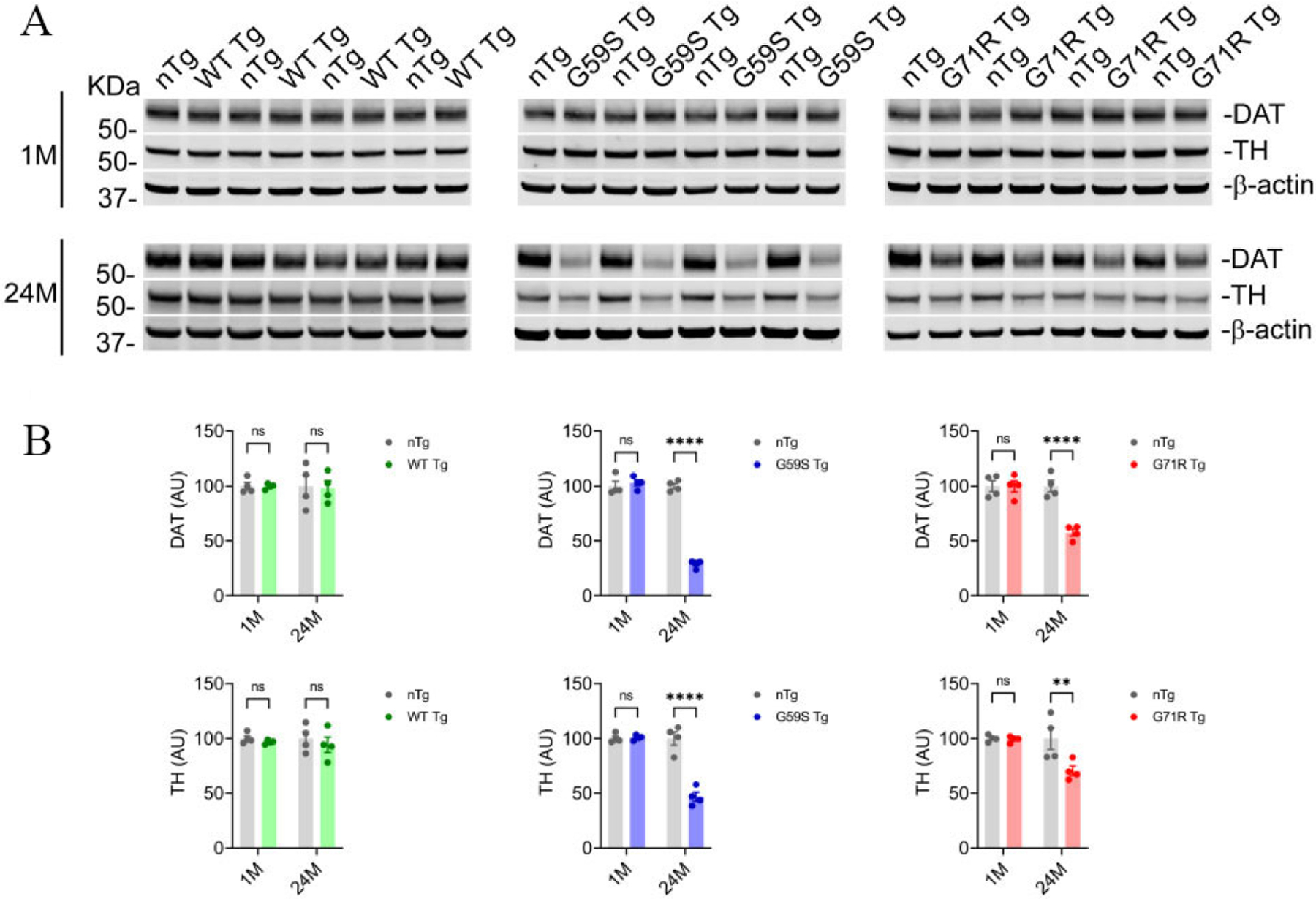
Reduction of dopaminergic proteins in the striatum of *G59S Tg* and *G71R Tg* mice. (A, B) Western blots show the expression of DAT and TH in the striatum homogenates of 1- and 24-month-old *nTg*, *WT Tg*, *G59S Tg*, and *G71R Tg* mice. Actin was used as the loading control. The bar graph estimates the levels of DAT and TH expression normalized against β-actin (*n* = 4 per genotype). Data are presented as mean ± SEM. Two-way ANOVA with Sidak’s multiple comparisons test was used for statistical analysis. **P = 0.0054, ****P < 0.0001. ns: Not significant.

Tyrosine hydrolase (TH) is the rate-limiting enzyme in dopamine synthesis, and dopamine transporter (DAT) mediates dopamine uptake^[32,33]^. Since TH and DAT play critical roles in the nigrostriatal DA pathway, the protein levels of TH and DAT in the striatal homogenates of 1- and 24-month-old *nTg*, *WT Tg*, *G59S Tg*, and *G71R Tg* mice were examined. At one month of age, *nTg*, *WT Tg*, *G59S Tg*, and *G71R Tg* mice had comparable levels of TH and DAT in the striatum [Figure 6A and B]. At 24 months of age, *WT Tg* mice showed no marked change in TH and DAT protein expression compared with nTg controls, while both *G59S Tg* and *G71R Tg* mice displayed substantial loss of TH and DAT [Figure 6A and B]. Interestingly, the percentages of DAT loss in the striatum of 24-month-old *G59S Tg* and *G71R Tg* mice (approximately 70% and 42%, respectively) were more severe than the TH loss (about 53% and 29%, respectively) [Figure 6A and B]. These data suggest expression of either G59S or G71R mutant p150^Glued^ in midbrain DA neurons may preferentially downregulate the expression and function of DAT protein at DA axon terminals.

### G59S Tg mice display age-dependent accumulation of p150^Glued^ aggregates in midbrain dopaminergic neurons

**Figure 7.**
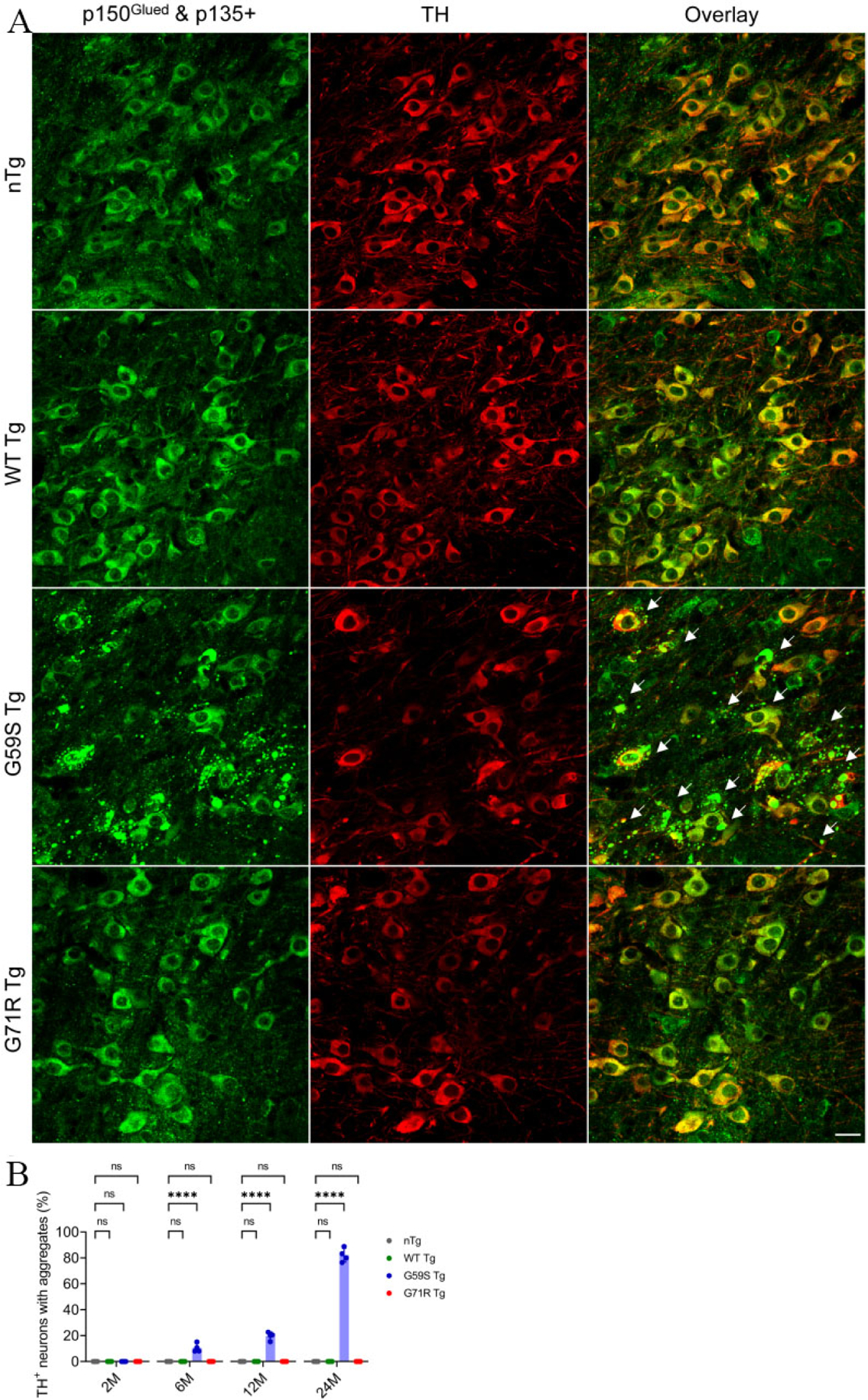
Formation of p150^Glued^-positive protein aggregates in midbrain DA neurons of *G59S Tg* mice. (A) Immunofluorescent images show the staining of p150^Glued^ and p135+ (green) and TH (red) in midbrain coronal sections of 24-month-old *nTg*, *WT Tg*, *G59S Tg*, and *G71R Tg* mice. Scale bar: 20 μm. Arrows point to proteins aggregates of p150^Glued^ and p135+ in the soma and neurites of DA neurons from *G59S Tg* mice. (B) Bar graph estimates the percentage of TH-positive neurons with proteins aggregates of p150^Glued^ and p135+ in the midbrain of 2-, 6-, 12-, and 24-month-old *nTg*, *WT Tg*, *G59S Tg*, and *G71R Tg* mice (at each time point, *n* = 4 animals per genotype and 5 sections per animal). Aggregates were defined as puncta ≥ 1 μm^2^. Data are presented as mean ± SEM. At each time point, one-way ANOVA with Dunnett’s multiple comparisons test was used for statistical analysis (the mean of each genotype was compared with the mean of nTg). ****P < 0.0001. ns: Not significant. TH: Tyrosine hydroxylase.

**Figure 8.**
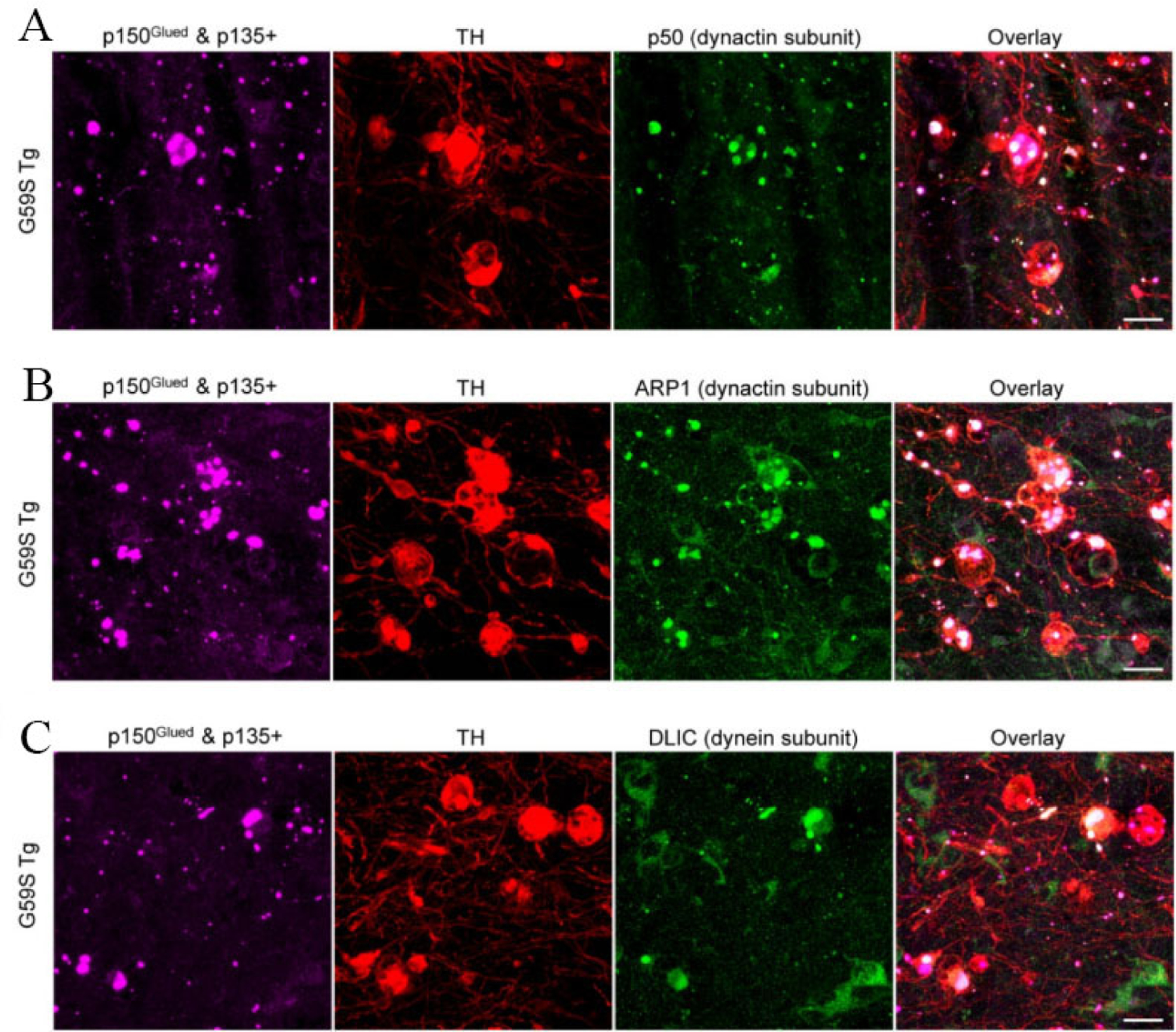
Co-aggregation of dynactin and dynein subunits with G59S mutation-induced p150^Glued^ aggregates in the dystrophic DA neurites of *G59S Tg* mice. (A) Immunofluorescent images show the staining of p150^Glued^ and p135+ (purple), TH (red), and dynactin subunit p50 (green) in the SNr of six-month-old *G59S Tg* mice. Scale bar: 20 μm. (B) Immunofluorescent images show the staining of p150^Glued^ and p135+ (purple), TH (red), and dynactin subunit ARP1 (green) in the SNr of six-month-old *G59S Tg* mice. Scale bar: 20 μm. (C) Immunofluorescent images show the staining of p150^Glued^ and p135+ (purple), TH (red), and dynein subunit DLIC (green) in the SNr of six-month-old *G59S Tg* mice. Scale bar: 20 μm.

**Figure 9.**
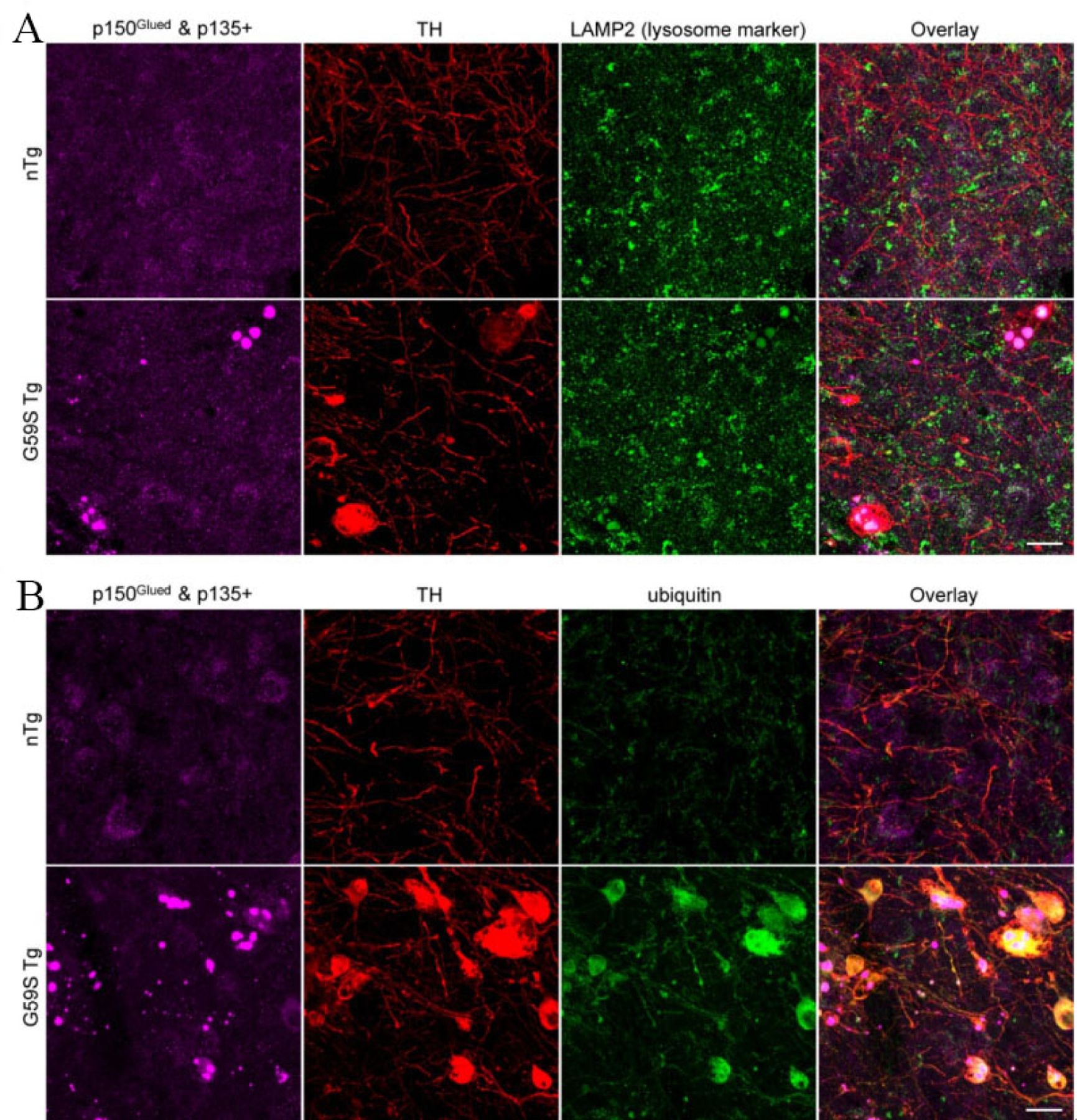
Co-localization of lysosomes with G59S mutation-induced p150^Glued^ aggregates and upregulated ubiquitination in the dystrophic DA neurites of *G59S Tg* mice. (A) Immunofluorescent images show the staining of p150^Glued^ and p135+ (purple), TH (red), and LAMP2 (green) in the SNr of six-month-old nTg and *G59S Tg* mice. Scale bar: 20 μm. (B) Immunofluorescent images show the staining of p150^Glued^ and p135+ (purple), TH (red), and ubiquitin (green) in the SNr of six-month-old *nTg* and *G59S Tg* mice. Scale bar: 20 μm.

As both G59S and G71R mutant p150^Glued^ were found aggregation-prone *in vitro*^[17–19]^, the subcellular distribution pattern of p150^Glued^ in the midbrain DA neurons of 2-, 6-, 12-, and 24-month-old *nTg*, *WT Tg*, *G59S Tg*, and *G71R Tg* mice was examined. TH and p150^Glued^ co-staining revealed diffuse cytoplasmic localization of p150^Glued^ without apparent aggregate formation in the soma and neurites of midbrain DA neurons from both young and aged *nTg*, *WT Tg*, and *G71R Tg* mice [Figure 7A and B]. By contrast, *G59S Tg* mice presented with p150^Glued^-positive cytoplasmic aggregates, mainly in the soma and in the neurites of midbrain DA neurons [Figure 7A]. Furthermore, the percentage of DA neurons with p150^Glued^ aggregate in *G59S Tg* mice increased in an age-dependent manner, approximately 0%, 10.54%, 19.66%, and 82.17% at 2, 6, 12, and 24 months of age, respectively [Figure 7B]. Immunostaining further revealed the co-aggregation of dynactin subunit p50 and ARP1, as well as dynein subunit DLIC, with p150^Glued^ aggregates inside the dystrophic DA neurites in the SNr of six-month-old *G59S Tg* mice [Figure 8A–C]. In addition, co-staining of coronal midbrain sections showed co-localization of lysosomes with p150^Glued^ aggregates and a substantial increase of ubiquitin immunoreactivities within the dystrophic DA neurites in the SNr of six-month-old *G59S Tg* mice, compared with nTg controls [Figure 9A and B]. Thus, G59S mutation rather than G71R mutation induces mutant p150^Glued^ to form cytoplasmic aggregates in midbrain DA neurons *in vivo*. Moreover, the aggregates trap dynein/dynactin, co-localize with lysosomes, and upregulate ubiquitination, implying detrimental effects of G59S mutant p150^Glued^ on dynein/dynactin function and the degradative pathway in the midbrain DA neurons. These findings may explain why *G59S Tg* mice developed severe and early-onset degeneration of midbrain DA neurons, while *G71R Tg* mice showed milder and more slowly progressive neurodegeneration.

## DISCUSSION

In this study, transgenic mouse lines with selective overexpression of wild-type human p150^Glued^, motor neuron disease-related G59S mutant p150^Glued^, or Perry syndrome-related G71R mutant p150^Glued^ in midbrain DA neurons were generated and characterized. The *WT Tg* mice did not develop any overt motor phenotypes or midbrain DA neurodegeneration compared with age-matched *nTg* mice. The *G59S Tg* mice displayed weight loss, motor deficits, early-onset decreased dopamine transmission, and early-onset progressive loss of DA neurons and axons. The *G71R Tg* mice exhibited hyperactivities, impaired motor coordination, early-onset increased dopamine release and slower dopamine uptake, and late-onset loss of DA neurons and axons. Furthermore, both *G59S Tg* and *G71R Tg* mice showed an age-dependent reduction of DA proteins in the striatum, with a more severe loss of DAT than TH. Finally, G59S mutant p150^Glued^ rather than G71R mutant p150^Glued^ was found to form aggregates in mouse midbrain DA neurons in an age-dependent manner, and the aggregates trapped dynein/dynactin, co-localized with lysosomes, and upregulated ubiquitination.

Since the initial identification of motor neuron disease- and Perry syndrome-causative mutations in p150^Glued^, several mouse strains of mutant p150^Glued^ have been generated to study their effects on selective degeneration of specific types of neurons. Motor neuron disease-like motor phenotypes and degeneration of spinal motor neurons have been reported in G59S p150^Glued^ transgenic and knock-in mouse models^[20–22]^. Decreased exploratory activity and impaired motor coordination were observed in the transgenic mice overexpressing G71A p150^Glued^ under the control of the *Thy1* promotor^[23]^. However, neither the expression of G71A p150^Glued^ in midbrain DA neurons nor neuronal loss in the brain tissue was examined in the transgenic mice^[23]^. Recently, G71A p150^Glued^ knock-in mouse models were created^[24]^. Increased immobility time in the tail suspension test, impaired motor coordination in the beam-walking test, and decreased TH staining intensity in the substantia nigra neurons were found in the heterozygous G71A p150^Glued^ knock-in mice^[24]^, recapitulating some of the clinical features of Perry syndrome. However, the pathogenic effects of mutant p150^Glued^ on Perry syndrome-related degeneration of the nigrostriatal DA pathway remain to be determined. New lines of transgenic mice (*WT Tg*, *G59S Tg*, and *G71R Tg*) with selective overexpression of wild-type, G59S mutant, or G71R mutant human p150^Glued^ in midbrain DA neurons were generated in the current study, allowing us to systematically evaluate the impact of mutant p150^Glued^ on survival and function of midbrain DA neurons. *WT Tg* mice showed neither motor phenotypes nor pathological, functional, or biochemical abnormalities of the nigrostriatal DA pathway. *G59S Tg* mice displayed substantial weight loss, dramatic reduction of rearing movement in the open field test, and significant impairment of motor coordination in the rotarod test. The pathophysiological basis for the motor phenotypes in *G59S Tg* mice is the early-onset and severe loss of DA neurons and axon terminals and the resultant deficiency of dopamine content and transmission. *G71R Tg* mice exhibited significant hyperactivities in ambulatory and rearing movements and substantially impaired rotarod performance through 6–18 months of age. In contrast to the severe and early-onset degeneration of nigrostriatal DA pathway in *G59S Tg* mice, the neurodegeneration in *G71R* mice was much milder and more slowly progressive, with no significant loss of DA neurons and axon terminals occurring before 18 months of age. Despite no early-onset DA neuron degeneration, *G71R Tg* mice showed an early-onset increase of dopamine release and slower dopamine uptake in the striatum, which may elevate the extracellular dopamine level and contribute to hyper-dopamine motor phenotypes.

Thus, our data demonstrate that both G59S and G71R mutant p150^Glued^ are pathogenic to the nigrostriatal DA pathway *in vivo*, but differentially impair the survival and functions of midbrain DA neurons. The finding on G59S mutation is unexpected since the mutation was particularly linked to a slowly progressive autosomal dominant form of lower motor neuron disease, the distal hereditary motor neuropathy 7B (HMN7B)^[6]^. Our previous study showed that both homozygous *Dctn1* knock-out mice and homozygous *DCTN1* G59S knock-in mice died during early embryogenesis, indicating the loss-of-function nature of G59S mutation^[20]^. Furthermore, we found that the G59S mutation disrupts the stability of p150^Glued^ protein, so we could not detect any expression and accumulation of p150^Glued^ protein in the tissues of homozygous *DCTN1* G59S knock-in embryos^[20]^. By contrast, in the *G59S Tg* mice, an artificial overexpression of G59S mutation in DA neurons led to an extensive accumulation of misfolded p150^Glued^ proteins, which may overwhelm the protein degradation system in the DA neurons and eventually induce cell death. Therefore, the overexpression data in either cell or animal models must be interpreted with caution.

Microtubule-binding domains in p150^Glued^ are required for the initiation of retrograde axonal transport, stability of axonal microtubules, and integrity of axon terminals^[13–16]^. Accordingly, degeneration of ventral root axons and disruption of neuromuscular junctions have been observed in G59S mutant p150^Glued^ transgenic mice^[21,22]^. In line with these earlier findings, the current study revealed that expression of either G59S or G71R mutant p150^Glued^ in midbrain DA neurons resulted in substantial dysfunction and degeneration of DA axon terminals in the striatum. Additionally, our study showed expression of either G59S or G71R mutant p150^Glued^ in midbrain DA neurons led to the dystrophy of DA neurites in the SNr, indicating an essential role of p150^Glued^ in maintaining the structural integrity of both axons and dendrites of DA neurons. The degeneration of norepinephrine neurons in locus coeruleus may be fundamentally linked to the loss of midbrain DA neurons by reducing brain norepinephrine and inducing chronic neuroinflammation^[36]^. The degeneration of serotonergic neurons in the medullary raphe and ventrolateral medulla may contribute to respiratory failure in Perry syndrome patients^[35]^. Future studies will be needed to determine the underlying mechanism of how mutant p150^Glued^ triggers the structural and functional changes of distal and proximal DA neurites.

Several mechanisms have been hypothesized to address the selective degeneration of motor neurons caused by G59S mutant p150^Glued^, including impaired retrograde axonal transport of trophic signaling, defects of vesicular trafficking in the ER-Golgi secretory pathway, aberrant autophagosome-lysosome degradative pathway, and cytotoxicity of mutant p150^Glued^ aggregates^[17,21,22]^. However, due to the lack of appropriate animal models, mechanisms for the selective degeneration of DA neurons in Perry syndrome have been poorly understood. Until recently, *DCTN1* G71A knock-in mice were generated, and homozygous knock-in mice died prenatally, suggesting a potential loss-of-function nature of the mutation^[24]^. In the heterozygous *DCTN1* G71A knock-in mice, a reduction of TH immunoreactivity in DA neurons was reported^[24]^. However, the effect of mutant p150^Glued^ on dopamine transmission and the related regulating proteins remains to be determined. Our HPLC and FSCV studies revealed that overexpression of human G59S p150^Glued^ in midbrain DA neurons led to substantial dopamine deficiency, significantly decreased dopamine release, and slower dopamine uptake, reflecting the severe and early-onset loss of DA neurons and axons. In contrast, overexpression of human G71R p150^Glued^ in midbrain DA neurons resulted in early-onset substantially increased dopamine release and slower dopamine uptake long before the loss of DA axons and the deficiency of dopamine content in the striatum occurred, indicating the early-onset dysfunction of DAT. Correlatively, Western blot analysis demonstrated that overexpression of either G59S or G71R mutant p150^Glued^ in midbrain DA neurons preferentially downregulated the expression of DAT at DA axon terminals. Further investigation will be required to unmask the specific cellular mechanisms leading to the DAT dysfunction and the selective cell death of DA neurons in Perry syndrome.

G59S mutation, as well as all the Perry syndrome-causative mutations, occurs within or close to the CAP-Gly domain of p150^Glued^, causing loss of function of the MTBDs^[6–8]^. G59S mutant p150^Glued^ is prone to misfold and form cytoplasmic aggregates leading to enhanced cell death both *in vitro* and *in vivo*, suggesting both loss-of-function and toxic-gain-of-function mechanisms are involved in G59S mutation-induced neurodegeneration^[17,21]^. Perry syndrome-related G71A mutant p150^Glued^ has been reported to aggregate less than G59S mutant p150^Glued^ when overexpressed in HeLa cells^[18]^. Moreover, no evident p150^Glued^ aggregates are observed in G71A transgenic mice^[23]^. In line with these earlier findings, our study revealed that neither wild-type nor G71R mutant p150^Glued^ formed aggregates *in vivo* when overexpressed in midbrain DA neurons. In contrast, G59S mutant p150^Glued^ formed aggregates in an age-dependent way when overexpressed in mouse midbrain DA neurons. Moreover, co-aggregation of dynactin and dynein subunits with G59S mutation-induced p150^Glued^ aggregates was observed inside the dystrophic DA neurites of *G59S Tg* mice. The data suggest the sequestration of active motors in aggregates; the restricted accessibility of adaptors, cargoes, and regulatory proteins to dynein/dynactin; and thus the impairment of normal functions of dynein/dynactin in the midbrain DA neurons. In addition, co-localization of lysosomes with G59S mutation-induced p150^Glued^ aggregates and upregulated ubiquitination were found within the dystrophic DA neurites of *G59S Tg* mice, implying the disruption of the degradative pathway in the midbrain DA neurons. These observations may explain why the degeneration of the nigrostriatal DA pathway in *G59S Tg* mice is more early-onset and severe than in *G71R Tg* mice in our study. Future studies will explore whether overexpression of exogenous G59S or G71R mutant p150^Glued^ compromises the normal functions of endogenous dynactin p150^Glued^ in the midbrain DA neurons to test if there is a dominant-negative mechanism for *DCTN1* mutation-induced degeneration of nigrostriatal DA pathway.

While the present study focused on the role of mutant p150^Glued^ in Perry syndrome-related parkinsonism and the underlying degeneration of midbrain DA neurons, the pathophysiological basis and pathogenic mechanisms of other Perry syndrome-related symptoms remain to be delineated in detail, including apathy/depression and central hypoventilation^[9,10]^. Except for the loss of midbrain DA neurons, Perry syndrome patients also display marked degeneration of norepinephrine neurons in *locus coeruleus* and serotonergic neurons in the medullary raphe and ventrolateral medulla^[34,35]^. Future studies will be needed to crossbreed our tetO-human *DCTN1* with suitable tTA lines and determine the pathogenic significance of mutant p150^Glued^ on norepinephrine neurons and serotonergic neurons.

While this study demonstrated the differential pathogenic impacts of G59S and G71R mutant p150^Glued^ on the survival and function of midbrain DA neurons, the underlying cellular and molecular mechanisms remain to be further investigated. Over the years, research has revealed that midbrain DA neurons are composed of diverse subsets with distinct molecular identities, connectivity, functionality, and vulnerability in Parkinson’s disease^[37]^. Identifying the selectively vulnerable subsets of midbrain DA neurons in Perry syndrome using our p150Glued transgenic mouse models would be an interesting area for future exploration. Apart from neuronal loss, TAR DNA-binding protein 43 (TDP-43) pathology is another neuropathological feature of Perry syndrome^[34]^. Future studies will be required to detect the cytoplasmic TDP-43 aggregates in the midbrain DA neurons of our transgenic mice.

In summary, our findings show selective expression of either G59S or G71R mutant p150^Glued^ in mouse midbrain DA neurons leads to parkinsonism-like motor abnormalities and degeneration of the nigrostriatal DA pathway, recapitulating some key clinical and pathological features of Perry syndrome. Moreover, G59S and G71R mutant p150^Glued^ exhibit differential pathogenic impact on the survival and function of midbrain DA neurons *in vivo*. The p150^Glued^ transgenic moue models provide valuable tools for mechanistic insights and therapeutic targets of DA neuron degeneration in Perry syndrome.

## Data Availability

Data and materials of the current study will be available upon reasonable request.
